# CaMKII-dependent regulation of cardiac Na^+^ homeostasis

**DOI:** 10.3389/fphar.2014.00041

**Published:** 2014-03-10

**Authors:** Eleonora Grandi, Anthony W. Herren

**Affiliations:** Department of Pharmacology, University of California at DavisDavis, CA, USA

**Keywords:** CaMKII, Na^+^ channel, DADs, Na^+^ overload, arrhythmia

## Abstract

Na^+^ homeostasis is a key regulator of cardiac excitation and contraction. The cardiac voltage-gated Na^+^ channel, Na_V_1.5, critically controls cell excitability, and altered channel gating has been implicated in both inherited and acquired arrhythmias. Ca^2^^+^/calmodulin-dependent protein kinase II (CaMKII), a serine/threonine kinase important in cardiac physiology and disease, phosphorylates Na_V_1.5 at multiple sites within the first intracellular linker loop to regulate channel gating. Although CaMKII sites on the channel have been identified (S516, T594, S571), the relative role of each of these phospho-sites in channel gating properties remains unclear, whereby both loss-of-function (reduced availability) and gain-of-function (late Na^+^ current, I_Na__L_) effects have been reported. Our review highlights investigating the complex multi-site phospho-regulation of Na_V_1.5 gating is crucial to understanding the genesis of acquired arrhythmias in heart failure (HF) and CaMKII activated conditions. In addition, the increased Na^+^ influx accompanying I_Na__L_ may also indirectly contribute to arrhythmia by promoting Ca^2^^+^ overload. While the precise mechanisms of Na^+^ loading during HF remain unclear, and quantitative analyses of the contribution of I_Na__L_ are lacking, disrupted Na^+^ homeostasis is a consistent feature of HF. Computational and experimental observations suggest that both increased diastolic Na^+^ influx and action potential prolongation due to systolic I_Na__L_ contribute to disruption of Ca^2^^+^ handling in failing hearts. Furthermore, simulations reveal a synergistic interaction between perturbed Na^+^ fluxes and CaMKII, and confirm recent experimental findings of an arrhythmogenic feedback loop, whereby CaMKII activation is at once a cause and a consequence of Na^+^ loading.

## CARDIAC Na^+^ HANDLING

**FIGURE 1:**
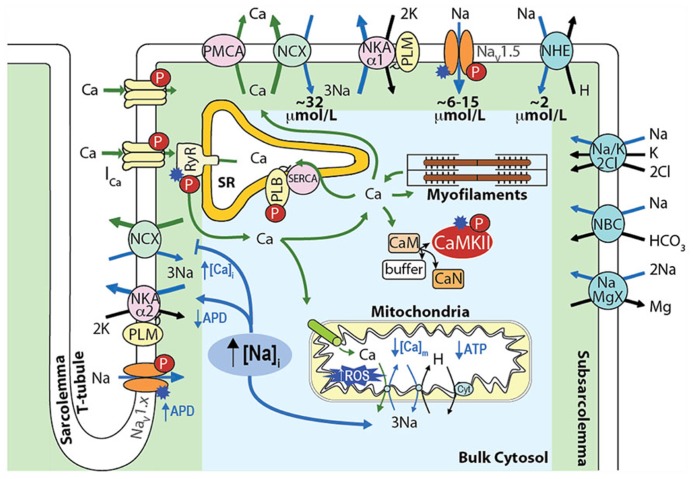
**Schematic showing the main processes regulating [Na^+^]_**i**_ and [Ca^**2**^^+^]_**i**_ homeostasis in cardiac myocytes and the mechanisms by which an increase in [Na^+^]_**i**_ affects [Ca^**2**^^+^]_**i**_, contractility, and metabolism.** Upon cardiac myocyte electrical excitation, Na_V_ opening initiates the AP upstroke and allows Na^+^ entry (limited to 6–15 μmol/L by rapid inactivation). E_m_ depolarization causes LTCC openings and consequent Ca^2^^+^-induced SR Ca^2^^+^ release, which triggers contraction. During relaxation, Ca^2^^+^ is reuptaken into the SR by SERCA, and extruded out of the cytosol through NCX, which extrudes the ~10 μmol/L Ca^2^^+^ that enters via LTCCs and leads to an increase in [Na^+^]_i_ by ~32 μmol/L during each AP. NHE brings in ~2 μmol/L Na^+^ at physiological intracellular pH (~16 μmol/L during intracellular acidosis). The Na^+^/HCO_3_^-^ and the Na^+^/K^+^/2Cl^-^ cotransporters, and the Na^+^/Mg^2^^+^ exchanger contribute minimally to the total Na^+^ entry (~40–45 μmol/L), which is then extruded by NKA [reviewed in (Despa and Bers, 2013)].

In cardiac myocytes, intracellular Na^+^ concentration ([Na^+^]_i_) is a key modulator of Ca^2^^+^ cycling, contractility and metabolism, and is controlled by the balance between Na^+^ influx and extrusion (**Figure 1**). The major contributors to Na^+^ entry during the cardiac cycle are the Na^+^/Ca^2^^+^ exchanger (NCX), the voltage-dependent Na^+^ channel (Na_V_), and the Na^+^/H^+^ exchanger (NHE) with relative contributions of NCX > Na_V_ > NHE (see **Figure 1**). Smaller amounts of Na^+^ also enter the cell via the Na^+^/HCO_3_^-^ and the Na^+^/K^+^/2Cl^-^ cotransporters, and the Na^+^/Mg^2^^+^ exchanger. Na^+^ efflux is controlled primarily through the Na^+^/K^+^ ATPase (NKA) that keeps [Na^+^]_i_ constant at steady-state [reviewed in (Despa and Bers, 2013)].

An integrative approach to cellular Na^+^ handling is critical to understand how these pathways interact spatially and temporally to affect cardiac cell function. Indeed, compelling evidence has accumulated that local [Ca^2^^+^]_i_ and [Na^+^]_i_ gradients exist close to the cell membrane (Carmeliet, 1992) that depend on the spatial localization of specific Na^+^ and Ca^2^^+^ handling proteins and their isoforms (**Figure 1**). For example, NKA-α2 is more concentrated at the t-tubules (whereas NKA-α1 is homogenously distributed), and this could be important in regulating local cleft [Na^+^]_i_ and [Ca^2^^+^]_i_ (Berry et al., 2007; Despa and Bers, 2007). NCX is concentrated at the t-tubules, but only a small NCX fraction colocalizes with proteins specific to the dyadic cleft [i.e., L-type Ca^2^^+^ channels (LTCCs) and ryanodine receptors (RyRs; Jayasinghe et al., 2009)]. Nevertheless, functional data indicate that NCX senses an early and high rise in local vs. bulk [Ca^2^^+^]_i_ (Weber et al., 2003a) and Ca^2^^+^ entry via reverse mode NCX can even trigger sarcoplasmic reticulum (SR) Ca^2^^+^ release (Litwin et al., 1998). Moreover, a loss-of-function mutation in ankyrin-B results in reduced NKA and NCX protein levels and impaired targeting to the t-tubules, which causes altered Ca^2^^+^ signaling and after contractions (Mohler et al., 2003) and can affect local [Na^+^], cellular and SR Ca^2^^+^ cycling (Camors et al., 2012), and kinase/phosphatase balance (DeGrande et al., 2012).

Cardiac Na^+^ channel (Na_V_1.5) activity also requires proper sarcolemmal localization. For example, defective membrane targeting by impaired interaction of Na_V_1.5 and ankyrin-G causes Brugada syndrome (BrS; Mohler et al., 2004). Na_V_1.5 are found roughly homogenously distributed in the t-tubules and external sarcolemma, whereas non-cardiac Na_V_ isoforms (whose role is still poorly understood) are more concentrated at the t-tubules (Brette and Orchard, 2006). However, immunofluorescence studies show Na_V_1.5 mostly at the intercalated disks, and it has been proposed that different pools of Na^+^ channels are located in the external sarcolemma vs. intercalated disks, where they interact within different macromolecular complexes and are regulated differently (Lin et al., 2011; Petitprez et al., 2011; Sato et al., 2011).

Altered Na^+^ homeostasis through the mechanisms described above (and in **Figure 1**) contributes to action potential (AP) and [Ca^2^^+^] cycling dysregulation, leading to arrhythmia, metabolic imbalance, remodeling and cell death, and is a hallmark of various cardiac diseases. The search for a common denominator has pointed to the Ca^2^^+^/calmodulin-dependent protein kinase II (CaMKII). CaMKII is a basophilic serine/threonine kinase that plays critical roles in cardiac physiology and disease (where it is often found hyperactive) through phosphorylation of several Ca^2^^+^ handling proteins and ion channels (**Figure 1**; Bers and Grandi, 2009; Anderson et al., 2011). Na_V_1.5, in particular, has emerged as a principle CaMKII target. Herein, we summarize growing evidence indicating an important role for CaMKII in regulating Na_V_1.5 gating and cardiomyocyte Na^+^ homeostasis, with an emphasis on the important interconnection between Na^+^ and Ca^2^^+^ handling, and consequences for arrhythmia.

## CARDIAC Na^+^ CHANNEL STRUCTURE AND FUNCTION

**FIGURE 2:**
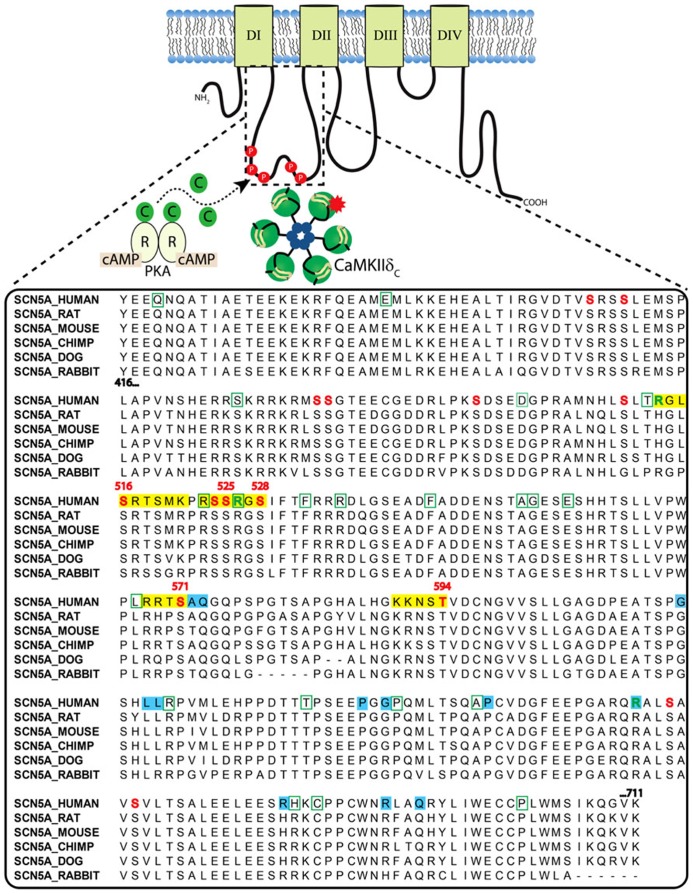
**Clustal sequence alignment of Na_**V**_1.5 I–II loop across indicated species.** Sites are color coded as follows: red font = phosphorylation; green font = methylation; yellow block = basophilic kinase consensus region; green bounding box = missense BrS mutation; blue block = LQTS mutation.

The cardiac Na^+^ channel is responsible for the upstroke of the cardiac AP and is a critical determinant of cardiac electrical excitability. The pore forming α-subunit, Na_V_1.5, is composed of four core domains (DI–DIV), each containing six transmembrane segments (S1–S6; ~230 kD; **Figure 2**). The positively charged S4 segments serve as the channel voltage sensors and the S5–S6 segments comprise the ion-conducting pore. Upon depolarization, the channel rapidly activates (<1 ms) producing a large (tens of nA), transient inward Na^+^-current, I_Na_, and then undergoes fast inactivation (within a few ms) through interactions of the well-described DIII–IV linker IFM motif with the pore (similar to N-type inactivation in K^+^ channels). This is followed by poorly understood slower modes of inactivation (hundreds of ms to a few s) that likely involve rearrangements of the pore structure (similar to C-type inactivation in K^+^ channels). Channels that fail to completely inactivate, or close and then reopen, give rise to a persistent, or late Na^+^ current (I_NaL_). Na_V_1.5α forms macromolecular complexes with β-subunits (β1 to β4) and many other accessory and regulatory proteins that modify channel gating [extensively reviewed by Abriel (2010); Wilde and Brugada (2011)]. The intracellular N- and C-termini and linker loops connecting DI–IV are also all involved in channel gating. In particular, the Na_V_1.5 I–II cytoplasmic linker loop is the subject of extensive post-translational regulation, and this region has received much attention as a hot spot for phosphorylation by CaMKII [reviewed in (Herren et al., 2013)].

## PHOSPHORYLATION OF Na_V_1.5 BY CaMKII

CaMKII activation has been shown to alter the gating properties of the Na^+^ channel, as summarized in **Table 1**. Specifically, CaMKII phosphorylation of Na_V_1.5 shifts the voltage dependence of inactivation to negative potentials without affecting channel activation, slows recovery from and enhances entry into slower forms of inactivation, and increases I_NaL_ (Wagner et al., 2006).

**Table 1:** Na_**V**_1.5 phosphorylation sites, their associated kinases, and biophysical effects.

Residue	Kinase	SSI	I_NaL_	Comments	Reference
S516	CaMKII	←	↔		Ashpole et al. (2012)
T594	CaMKII	←	↔		Ashpole et al. (2012)
S571	CaMKII	←	↑	Basally phosphorylated by MS	Hund et al. (2010), Koval et al. (2012), Marionneau et al. (2012)
S525	PKA	←	ND	EP effects are indirect; mutant EP studies ND	Zhou et al. (2000)
S528	PKA	←	ND	EP effects are indirect; mutant EP studies ND	Zhou et al. (2000)
S457, S460, S483/4, S497, S510, S524/5, S664, S667	ND	ND	ND	MS identification; basal phosphorylation in untreated mouse heart lysates; responsible kinases ND	Marionneau et al. (2012)

*EP = electrophysiological; ND = not determined; MS = mass spectrometry.*

The Na_V_1.5 I–II linker loop interacts with CaMKII (Ashpole et al., 2012) and contains multiple basophilic kinase consensus sequences. Mass spectrometry analysis of wild type Na_V_1.5 (purified from mouse heart lysates) revealed several sites within this loop that are basally phosphorylated (Marionneau et al., 2012; **Figure 2**, red color, and **Table 1**). Hund et al. (2010) mutated putative CaMKII consensus sites to non-phosphorylatable alanine residues and determined that CaMKII specifically phosphorylates S571. When expressed in a heterologous expression system, the S571A non-phosphorylatable mutant abolished constitutively active co-expressed CaMKII enhancement of channel inactivation and I_NaL_. On the other hand, phosphomimetic S571E recapitulated CaMKII effects (but still in the presence of constitutively active CaMKII). S571 phosphorylation was increased in murine (Toischer et al., 2013) and human heart failure (HF) and canine ischemic cardiomyopathy (Koval et al., 2012).

Because CaMKII (and other kinases) can phosphorylate non-canonical sequences, we screened the entire Na_V_1.5 I–II loop for CaMKII phosphorylation with an *in vitro* kinase assay system (Ashpole et al., 2012). This assay consisted of a tiled peptide array spanning the entire loop region followed by *in vitro* phosphorylation with recombinant CaMKII and revealed that site S516 was phosphorylated most efficiently by CaMKII (Ashpole et al., 2012). Immobilized peptides containing S483/S484 and S593/T594 were also phosphorylated in this assay at much lower efficiency. It is important to consider peptide conformation may change when a peptide is immobilized or soluble, which can affect kinase access to phospho-acceptor sites (Bayer et al., 2006). In fact, subsequent studies using soluble peptides showed that T594 but not S483/4 could be phosphorylated, although only at low efficiencies. Furthermore, peptide conformation and kinase binding may be different for the full-length channel, thus affecting its ability to be phosphorylated. Therefore, additional phosphorylation studies using full-length Na_V_1.5 are needed.

Wild type or non-phosphorylatable mutant channels (S516A, T594A, S571A) were coexpressed with CaMKIIδC in HEK293 cells and voltage-clamped under pipette conditions to acutely activate CaMKII (with Ca^2^^+^ and calmodulin), with or without CaMKII inhibition [by autocamtide-2 inhibitory peptide (AIP); Ashpole et al., 2012]. CaMKII shifted steady-state inactivation (SSI) to hyperpolarizing potentials and increased entry into inactivation, and this was blocked with AIP or by mutating these sites to non-phosphorylatable alanine. Phosphomimetic S516E and T594E mutants recapitulated the hyperpolarizing shift in SSI even in the absence of CaMKII and presence of AIP. In our hands, however, S571E phosphomimetic mutants showed no statistically significant negative shift in SSI. Moreover, we observed no enhancement of late I_NaL_ in any of the mutants tested, but we did not coexpress β-subunits, which some have indicated to be important for I_NaL_ (Maltsev et al., 2009). Importantly, the phosphorylation status of S516 and T594 in native myocytes is yet to be determined, and more studies are needed to determine the relative contribution of these sites to specific channel gating properties.

## FUNCTIONAL CONSEQUENCES OF I–II LOOP PHOSPHORYLATION: INSIGHT FROM INHERITED MUTATIONS

Structure-function studies of *SCN5A* channelopathies/inherited mutations may further an understanding of the functional consequences of Na_V_1.5 phosphorylation. CaMKII-dependent alterations of Na_V_1.5 gating are remarkably similar to those caused by the Na_V_1.5 mutation 1795insD (Bezzina et al., 1999), which is associated to patients with mixed long QT syndrome (LQTS) and BrS phenotypes. DelK1500 is another overlap mutation that causes both loss and gain of function channel effects and is functionally similar to the effects of CaMKII phosphorylation on Na_V_1.5 (Grant et al., 2002). Experiments and simulations have shown how the heart rate acts as a switch imparting LQTS or BrS phenotypes to the same genotype (Veldkamp et al., 2000; Clancy and Rudy, 2002), and we have hypothesized a similar scenario for CaMKII effects (Grandi et al., 2007). However, 1795insD and delK1500 are present on the Na_V_1.5 C-terminus and III–IV loop respectively, making structural correlation with CaMKII phosphorylation on the I–II loop difficult. To date, no overlap mutations have been identified anywhere within the I–II loop (Remme et al., 2008).

While no overlap mutations are present, several mutations or polymorphisms have been identified within the I–II loop phosphorylation hot spot through studies of LQTS and BrS patient cohorts. Examination of these mutations may be useful in dissecting out the structure-function relationship of kinase phosphorylation within this region. More than 30 putative BrS mutations have been identified in the I–II loop from residues 416–711 (Kapplinger et al., 2010; **Figure 2**). Unfortunately, not all of these have been followed up with functional studies. One mutation, L567Q, was identified in a family exhibiting BrS and sudden infant death syndrome (Priori et al., 2000). When expressed heterologously, the mutant Na_V_1.5 channel displayed a negative shift in inactivation and enhanced entry into inactivation that was not dependent on coexpression of β-subunits (Wan et al., 2001). Another mutation, T512I, was identified in a patient exhibiting cardiac conduction disease, and resulted in hyperpolarizing shifts in SSI and activation and enhanced slow inactivation (Viswanathan et al., 2003). Not only is this mutation juxtaposed to one of the known CaMKII phosphorylation consensus regions identified at R513 and phosphorylated at S516 [see **Figure 2** and (Ashpole et al., 2012)], it also functionally mirrors the enhanced inactivation conferred by CaMKII phosphorylation at S516. Furthermore, PKA phosphorylation of nearby S525 and S528 (Murphy et al., 1996) has been previously described to similarly shift the voltage dependence of inactivation to negative potentials (Zhou et al., 2000). Thus, phosphorylation by either PKA (S525, S528) or CaMKII (S516) within a short ~10 amino acid stretch results in similar channel biophysics compared with the loss of function BrS mutation identified at T512. Moreover, a recent proteomics study demonstrated that residues R513 and R526 within this same region can be methylated, but the functional effect of this post-translational modification is unknown (Beltran-Alvarez et al., 2011). Another mutation, G514C, was identified in a family with cardiac conduction disease. Under voltage clamp, this mutation displayed a mixed biophysical phenotype of destabilized inactivation and decreased activation (Tan et al., 2001). These observations cement the importance of this short region in voltage-dependent inactivation of the channel.

CaMKII-dependent phosphorylation at S571 has been shown to result in both loss and gain of channel function (Hund et al., 2010). The functional effects of a negative charge at this site from CaMKII phosphorylation were suggested to phenocopy the adjacent inherited LQTS charge mutations at A572D and Q573E (Koval et al., 2012). Although A572D was initially identified as a causative LQTS mutation (Tester et al., 2005), a subsequent study showed that this mutation is actually a benign variant and does not cause LQTS in and of itself (Tester et al., 2010). Similarly, a mutation identified in a LQTS patient at 619 was also attributed to an observed LQTS phenotype in this patient. This L619F mutation was found to induce I_NaL_ when expressed in a heterologous expression system (Wehrens et al., 2003). However, a LQTS screening study from the Roden group failed to detect any late I_NaL_ (or gating changes) from L618F mutants expressed in heterologous cells and concluded this mutation is also a benign variant (Yang et al., 2002). Indeed, mutations within the Na_V_1.5 I–II loop have poor disease predictive value stemming from the high incidence of benign variants within this region (Kapa et al., 2009).

Thus, a clear and consistent role for the Na_V_1.5 I–II loop in loss of function inactivation gating emerges from studies of kinase phosphorylation (both CaMKII and PKA) and inherited mutations within this hotspot. There is less evidence for this loop region mediating I_NaL_ effects, with different groups providing contradictory results. It remains to be determined whether other unidentified CaMKII phospho-sites exist on other intracellular loops of Na_V_1.5 that may mediate (contribute to) enhancement of I_NaL_ (e.g., the C-terminus (Coyan et al., 2014) or III–IV loop where overlap mutations have been identified). It is also conceivable that I_NaL_ enhancement in pathological conditions could be due to an increase in neuronal Na^+^ channel isoforms with higher fractional I_NaL_ (Xi et al., 2009; Biet et al., 2012; Yang et al., 2012; Toischer et al., 2013). Alternatively, the effect of CaMKII to augment I_NaL_ may require accessory proteins not present in heterologous cell systems [such as regulatory β subunits (Maltsev et al., 2009)]. Indeed, this emphasizes the need for studies of these phosphorylation sites in native adult cardiomyocytes. Human induced pluripotent stem cells may also be used as suitable models, as has been done for some inherited mutations, such as 1795insD (Davis et al., 2012).

Enhanced inactivation and consequent reduction in channel availability due to CaMKII phosphorylation is expected to contribute to re-entrant arrhythmias from slowed conduction and enhanced dispersion of repolarization [as we have extensively reviewed previously (Herren et al., 2013)]. On the other hand, CaMKII enhancement of I_NaL_, while not as well understood mechanistically, can also lead to arrhythmias arising from prolonged AP duration (APD) that makes the cell more vulnerable to triggered activity [via early after-depolarizations (EADs)]. EADs are favored by conditions leading to prolongation of the AP plateau within a voltage range permitting recovery from inactivation and reactivation of LTCC, or can be the consequence of SR Ca^2^^+^ release and consequent augmentation of NCX current. A novel and unique mechanism underlying phase 3 EADs in ventricular myocytes from CaMKIIδC overexpressing mice has been recently described (Edwards et al., 2012), which involves isoproterenol-induced exaggerated Ca^2^^+^ release, increased inward NCX current, and non-equilibrium reactivation of fast I_Na_.

## I_NaL_ AND CELLULAR Na^+^ LOADING IN HF

**FIGURE 3:**
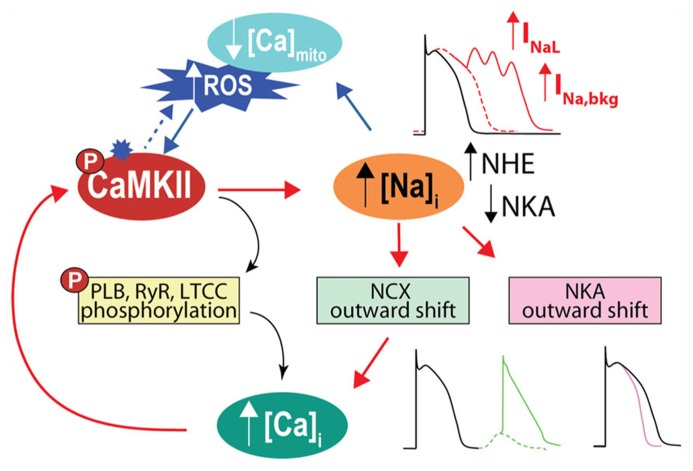
**CaMKII activation and Na^+^ homeostasis are intimately related in the regulation of cellular Ca^**2**^^+^, contractility and electrical stability.** With CaMKII hyperactivity, increase in late I_Na_ favors AP prolongation and EADs. Cellular Na^+^ loading increases Ca^2^^+^, via outward shift in NCX, which further activates CaMKII, fueling a vicious cycle that favors spontaneous SR Ca^2^^+^ release and predisposes to Ca^2^^+^-related arrhythmia. Elevated [Na^+^]_i_ can also influence ROS production by affecting mitochondrial [Ca^2^^+^]_i_. A less appreciated observation is that high [Na^+^]_i_ by causing outward shifts in both NCX and NKA will tend to shorten the AP, as predicted by computational AP models (Grandi et al., 2010, 2011). We speculate that the increased [Na^+^]_i_ in HF may limit AP prolongation caused by reduced K^+^ channel conductance and increased I_NaL_.

In addition to directly affecting myocytes electrical stability, I_Na_ is a major pathway of Na^+^ influx into cardiac myocytes, although NCX plays the most dominant role both at rest and in contracting cells [see **Figure 1** and (Despa and Bers, 2013)]. In normal myocytes I_Na__L_ contribution to Na^+^ entry is limited, but when I_NaL_ is enhanced in diseased conditions (such as cardiac hypertrophy and HF) it carries as much Na^+^ as the fast I_Na_ transient, thus increasing total cellular Na^+^ influx during a cardiac cycle and potentially contributing to Na^+^ overload (Makielski and Farley, 2006; Despa and Bers, 2013). Indeed, intracellular Na^+^ concentration ([Na^+^]_i_) is increased in myocytes from failing hearts compared to non-failing myocytes by 2–6 mM (Pogwizd et al., 2003; Shryock et al., 2013), but the precise mechanism(s) remains unclear (**Figure 3**). It has been suggested that CaMKII and enhanced I_NaL_ contribute to [Na^+^]_i_ elevation in HF, as CaMKIIδC overexpressing mice with HF exhibit prominent I_NaL_ and have increased [Na^+^]_i_ (Wagner et al., 2006).

While the increase in I_NaL_ may play a role in the observed [Na^+^]_i_ loading in these transgenic mice (Wagner et al., 2006), we demonstrated that CaMKII-dependent enhancement of I_NaL_ is not quantitatively sufficient to account for the [Na^+^]_i_ elevation observed in HF (Grandi et al., 2007; Wagner et al., 2011; Moreno et al., 2013; Morotti et al., 2014). Additionally, in a rabbit model of pressure- and volume-overload-induced HF, Despa et al. (2002) reported an increased Na^+^ influx resulting primarily from a TTX-sensitive pathway, which accounted for a gain in [Na^+^]_i_ of ~3 mM, and was present both in resting and electrically stimulated cells. This supports the existence of a diastolic Na^+^ influx in failing myocytes responsible for [Na^+^]_i_ elevation, although it is currently unclear whether this diastolic influx is carried by Na_V_s [whether they be of cardiac, neuronal, or skeletal muscle isoforms (Biet et al., 2012; Yang et al., 2012)] or by which gating mechanism. Increases of Na^+^ window and/or background current can potentially contribute to increased Na^+^ influx in failing vs. normal myocytes [and could be modified by drugs affecting the voltage dependence of Na^+^ channel gating (Shryock et al., 2013)]. We showed that simulation of an increased sarcolemmal Na^+^ leak current allows recapitulating the [Na^+^]_i_ gain seen in HF (Wagner et al., 2011; Moreno et al., 2013; Morotti et al., 2014). Baartscheer et al. (2003) using the same pressure- and volume-overload rabbit HF model in Despa et al. (2002) found that increased Na^+^ influx via NHE was the largest contributor to the elevated Na^+^ influx rate and [Na^+^]_i_ in paced myocytes. Interestingly, CaMKII activates NHE (Vila-Petroff et al., 2010). Reduced Na^+^ extrusion could also contribute to the observed intracellular Na^+^ gain during HF. Despite reduced expression, NKA function is unchanged in HF (Despa et al., 2002; Baartscheer et al., 2003), which is possibly due to higher NKA function secondary to reduced relative expression and elevated phosphorylation of phosholemman (Bossuyt et al., 2005).

## CONSEQUENCES OF Na^+^ LOADING ON EXCITATION-CONTRACTION COUPLING

[Na^+^]_i_ elevation in HF is expected to limit Ca^2^^+^ extrusion via forward mode NCX, and could even favor Ca^2^^+^ entry via reverse mode NCX (Weber et al., 2003b; **Figures 1** and **3**). Slowing Ca^2^^+^ extrusion via NCX will tend to elevate diastolic [Ca^2^^+^]_i_, thereby contributing to diastolic dysfunction. The slowed [Ca^2^^+^]_i_ decline and elevated diastolic [Ca^2^^+^]_i_ will also tend to increase SR Ca^2^^+^ content, thus exerting a positive inotropic effect and enhancing contractility. This explains the efficacy of cardiac glycosides in the treatment of congestive HF. These compounds promote inotropy by selectively inhibiting NKA and thereby impairing Na^+^ extrusion and weakening the NCX Ca^2^^+^ extrusion gradient. However, they are well known for having undesired arrhythmic consequences (Altamirano et al., 2006) by increasing the propensity for spontaneous SR Ca^2^^+^ release and delayed after-depolarizations (DADs). DADs arise from a transient inward current I_ti_ through forward mode NCX, which is evoked by the sudden increase in [Ca^2^^+^]_i_. It has been recently proposed that CaMKII is mechanistically involved in glycoside-induced arrhythmogenesis, as ouabain increased CaMKII activity, and CaMKII inhibition significantly reduced ouabain-induced spontaneous contractile activity and Ca^2^^+^ waves (Gonano et al., 2011). CaMKII overexpression exacerbated ouabain-induced spontaneous contractile activity (Gonano et al., 2011), possibly by favoring spontaneous SR Ca^2^^+^ release and DADs, as demonstrated in a recent computational mouse model (Morotti et al., 2014). Ouabain-induced [Na^+^]_i_ loading has also been shown to result in apoptosis (Sapia et al., 2010), mediated by reverse mode NCX-dependent activation of CaMKII. Thus, CaMKII inhibition may have potential therapeutic benefit during glycoside treatment to prevent arrhythmia and cell death while minimally impacting the positive inotropic effect. Analogously, the Na^+^ channel opener ATX-II induces spontaneous diastolic Ca^2^^+^ release from the SR and DADs in myocytes (Song et al., 2008) and arrhythmia in Langendorff perfused hearts (Yao et al., 2011), which is attenuated with CaMKII or I_NaL_ inhibition.

Our recent model of the failing human ventricular myocyte confirmed that a 20% increase in [Na^+^]_i_ in HF compared to control conditions slows forward mode inward NCX (Ca^2^^+^ extrusion) and enhances reverse mode NCX (Ca^2^^+^ entry, at the beginning of the AP; Moreno et al., 2013). This, coupled with AP prolongation due to extensive ionic remodeling in HF (including enhanced I_NaL_), increased diastolic [Ca^2^^+^]_i_ while maintaining adequate SR Ca^2^^+^ load and Ca^2^^+^ transient despite reduced SR Ca^2^^+^-ATPase (SERCA) function. However, [Na^+^]_i_-induced Ca^2^^+^ enhancement, in combination with hyperphosphorylated RyRs, causes diastolic SR Ca^2^^+^ release, I_ti_, and triggered APs [**Figure 3**, also favored by a more depolarized resting membrane potential (E_m_) in HF, due to decreased I_K1_ and increased background Na^+^ current]. Simulations showed that by targeting pathological late Na^+^ current and diastolic Na^+^ influx, ranolazine (1) limits [Na^+^]_i_ thus restoring normal NCX forward mode that speeds up Ca^2^^+^ extrusion, (2) shortens APD, thus further limiting Ca^2^^+^ entry, and (3) hyperpolarizes the E_m_, which elevates the threshold of triggered APs. These simulation results are consistent with recent experimental data in human failing myocytes from hypertrophic cardiomyopathy samples (Coppini et al., 2013). Similarly, CaMKII-dependent [Na^+^]_i_ elevation (normalized by ranolazine) has been associated with diastolic dysfunction and arrhythmias in CaMKIIδC overexpressing mice with HF (Sossalla et al., 2011). Taken together, these observations suggest that limiting [Na^+^]_i_ overload may be an appropriate antiarrhythmic therapeutic for the prevention of diastolic tension and arrhythmia triggers driven by [Ca^2^^+^]_i_ loading.

## ARRHYTHMOGENIC CaMKII–Na^+^–Ca^2^^**+**^–CaMKII FEEDBACK

It has been proposed that [Ca^2^^+^]_i_ loading caused by elevated [Na^+^]_i_ in HF may positively feed back to further activate CaMKII (and enhance target phosphorylation) thus creating an arrhythmogenic vicious circle. This positive feedback from Na^+^ to Ca^2^^+^ to CaMKII to Na^+^ has been qualitatively described for Na^+^ loading induced by ATX-II or accompanying a LQT3 mutation associated with increased I_NaL_ (Yao et al., 2011). Both of these conditions increased [Ca^2^^+^]_i_, induced CaMKII activation, increased CaMKII-dependent phosphorylation of phosholamban and RyRs, and favored arrhythmias. Although a CaMKII-dependent increase in Na_V_ phosphorylation was not directly confirmed in that study, a [Ca^2^^+^]_i_-dependent increase in I_NaL_ involving both CaMKII and PKC has previously been demonstrated (Ma et al., 2012). Accordingly, blockade of I_Na_ or I_NaL_ with TTX or ranolazine reversed all these effects, as did CaMKII inhibition via AIP or KN-93 (Yao et al., 2011). Our recently developed mouse myocyte model showed that alterations in Na^+^ handling accompanying transgenic CaMKII overexpression (namely, increase in systolic I_NaL_ and diastolic Na^+^ leak) can increase intracellular Na^+^ gain to initiate Ca^2^^+^ overload and CaMKII activation. Furthermore, this mechanism proved to be quantitatively sufficient to further disrupt Ca^2^^+^ (and Na^+^) homeostasis and promote cellular arrhythmias (Morotti et al., 2014). The simulated effect of Na^+^ loading to fuel CaMKII-Na^+^-Ca^2^^+^-CaMKII feedback was even more striking when CaMKII was further activated by isoproterenol. This is consistent with the observation that β-adrenergic stimulation of myocytes isolated from mice overexpressing CaMKII (Sag et al., 2009) or subjected to TAC-induced HF (Toischer et al., 2013) increased the number of DADs, which were largely prevented by either ranolazine or AIP (Toischer et al., 2013).

## CONSEQUENCES OF [Na^+^]_i_ LOADING ON CARDIAC ENERGETICS

[Na^+^]_i_ is also important in regulating cardiac myocyte bioenergetics, by controlling mitochondrial [Ca^2^^+^] via the mitochondrial NCX, and critically regulating the production of mitochondrial reactive oxygen species (ROS, **Figure 1**). High [Na^+^]_i_ in HF may negatively affect cardiac metabolism during rapid pacing, as elevated [Na^+^]_i_ has been shown to impair frequency-induced mitochondrial [Ca^2^^+^] accumulation thereby decreasing NADH/NAD^+^ redox potential, and increasing H_2_O_2_ formation in myocytes from failing hearts (Liu and O’Rourke, 2008). Notably, higher oxidative stress may further exacerbate [Na^+^]_i_ loading by enhancing I_NaL_ through direct effects on Na_V_1.5 or secondarily by activating CaMKII (Wagner et al., 2011). This suggests the interesting notion that ROS, like Na^+^, is both a cause and consequence of CaMKII activation. Not only does oxidative stress activate CaMKII directly (Erickson et al., 2008), but CaMKII-induced [Na^+^]_i_ loading can induce an increase in ROS (**Figures 1** and **3**). This parallels recent evidence indicating that CaMKII regulates ROS production (Pandey et al., 2011; Sepulveda et al., 2013; Zhu et al., 2014). Li et al. (2012) put forward an intriguing model-based hypothesis that Na^+^ accumulation in the SERCA knock-out mouse, which occurs secondary to marked NCX upregulation and intracellular acidosis, might play a role in the development of HF in these animals. This mechanism acts by initiating a reinforcing cycle involving a mismatch between ATP supply and demand, increasingly compromised metabolism, decreased intracellular pH, and further elevation of [Na^+^]_i_ due to NHE, which may shift the time of transition from compensated to decompensated function.

## CONCLUDING REMARKS

CaMKII-dependent loss and gain of function effects on Na_V_1.5 gating are pro-arrhythmic and functionally phenocopy *SCN5A* inherited mutations causing LQTS and BrS. Analogous to BrS mutations, CaMKII-dependent loss of Na^+^ channel function and consequent reduction in Na^+^ channel availability slows cardiac conduction and increases the propensity for conduction block and re-entry. Evidence points to the I–II loop phosphorylation hot spot as a likely mediator of these loss-of-function effects. Conversely, CaMKII enhancement of I_NaL_, while not as well understood, may prolong the APD and predispose to lethal ventricular tachyarrhythmia (as in LQTS). Further study of these mechanisms may provide important advances for rational design of novel antiarrhythmic therapeutics. Additionally, experimental data and quantitative findings support the intriguing hypothesis that drugs designed to correct aberrant Na^+^ channel gating behavior, causing I_NaL_ and cellular Na^+^ loading, may act importantly by reducing Ca^2^^+^ overload. Growing evidence suggests that prevention of Na^+^-dependent Ca^2^^+^ overload is a key mechanism of action for these compounds (Sossalla et al., 2011; Yao et al., 2011). Inhibition of intracellular Na^+^ loading can contribute to normalizing Ca^2^^+^ and E_m_ homeostasis in HF, but should not be achieved at the expense of systolic function. Inhibition of CaMKII or its targets may be an attractive means of facilitating these outcomes.

## AUTHOR CONTRIBUTIONS

Eleonora Grandi and Anthony W. Herren wrote the article.

## Conflict of Interest Statement

The authors declare that the research was conducted in the absence of any commercial or financial relationships that could be construed as a potential conflict of interest.
